# Defects Produced during Wet Transfer Affect the Electrical Properties of Graphene

**DOI:** 10.3390/mi13020227

**Published:** 2022-01-29

**Authors:** Dongliang Zhang, Qi Zhang, Xiaoya Liang, Xing Pang, Yulong Zhao

**Affiliations:** State Key Laboratory for Manufacturing Systems Engineering, School of Mechanical Engineering, Xi’an Jiaotong University, No. 28, Xianning West Road, Xi’an 710049, China; zhangdl666@stu.xjtu.edu.cn (D.Z.); liang_xiaoya@stu.xjtu.edu.cn (X.L.); px2014@stu.xjtu.edu.cn (X.P.)

**Keywords:** graphene, wet transfer technology, surface defects, electrical property, PMMA residue, lamination

## Abstract

Graphene has been widely used due to its excellent electrical, mechanical and chemical properties. Defects produced during its transfer process will seriously affect the performance of graphene devices. In this paper, single-layer graphene was transferred onto glass and silicon dioxide (SiO_2_) substrates by wet transfer technology, and the square resistances thereof were tested. Due to the different binding forces of the transferred graphene surfaces, there may have been pollutants present. PMMA residues, graphene laminations and other defects that occurred in the wet transfer process were analyzed by X-ray photoelectron spectroscopy and Raman spectroscopy. These defects influenced the square resistance of the produced graphene films, and of these defects, PMMA residue was the most influential; square resistance increased with increasing PMMA residue.

## 1. Introduction

Since the discovery of single-atom layers of graphene in 2004 [1,2], graphene and other two-dimensional materials have become a research focus. It was found that graphene has excellent electrical and mechanical properties [3,4,5,6,7,8,9]. Thus, graphene has been widely used in micro/nano devices. Various graphene pressure sensors with excellent performance have been successively proposed [10,11,12], as have been gas sensors based on graphene with different structures [13,14,15]. The excellent electrical properties of transferred graphene are the basis by which graphene sensors obtain good sensitivities. With the maturation of the chemical vapor deposition (CVD) method of preparing graphene [16], the transfer process also matured; yet there are still defects after transfer [17]. This study mainly explores the influence of defects in the graphene transfer process on its electrical properties, laying a foundation for obtaining graphene with even better electrical properties in the future. Single-layer graphene has a large specific surface area and is highly sensitive to the environment [18,19,20,21]. Therefore, the method by which graphene is transferred to a target substrate is crucial.

The PMMA-assisted transfer method is the most popular way to transfer graphene to a target substrate [22,23,24]. PMMA is spin-coated on CVD-grown graphene and cured by heating, after which the metal is etched away. The PMMA/graphene stack is transferred to the target substrate, and then the supportive PMMA is etched away by acetone [24,25]. Some drawbacks of this transfer process that affect the performance of the produced graphene include PMMA residues, cracks and tears [24,25,26,27,28,29]. Polymer residues are present in the transfer process [22,25], and the oxygen-containing functional groups of PMMA form p-type doping with graphene, reducing its electron mobility. Additionally, the adsorption or desorption of gas molecules in the air (e.g., NH_3_, H_2_O, NO_2_) on the graphene surface also change the local carrier density, resulting in step-like changes in resistance [13,30]. The sp^3^ content increases when placed in the air for a long time [31,32]. The square resistance of graphene is proportional to the number of graphene layers; thus, the occurrence of lamination in the transfer process multiplies the square resistance of the produced graphene [22].

In this paper, single-layer graphene is transferred to glass slides and SiO_2_ substrates by the wet transfer method, and the resistance of each sample is tested by a four-probe square resistance tester. Then, the surface roughness of the SiO_2_ substrate and graphene are measured by AFM to observe impurities in the produced graphene. The surface hydrophilicity is measured by a surface tension instrument to determine the binding force of the surface, characterizing the graphene’s surface quality. By X-ray energy spectrum analysis, the adsorbed elements of the representative samples on the slides and SiO_2_ substrates are determined. Additionally, Raman spectroscopy is used to analyze the surface spectra of two groups of samples with different representative resistances to study the layer distribution on the graphene surface after transfer. The experiment explores the influence of defects produced during the wet transfer on the obtained graphene’s electrical properties, laying a foundation for the subsequent improvement of these electrical properties.

## 2. Materials and Methods

### Graphene Transferring Process

**Figure 1:**
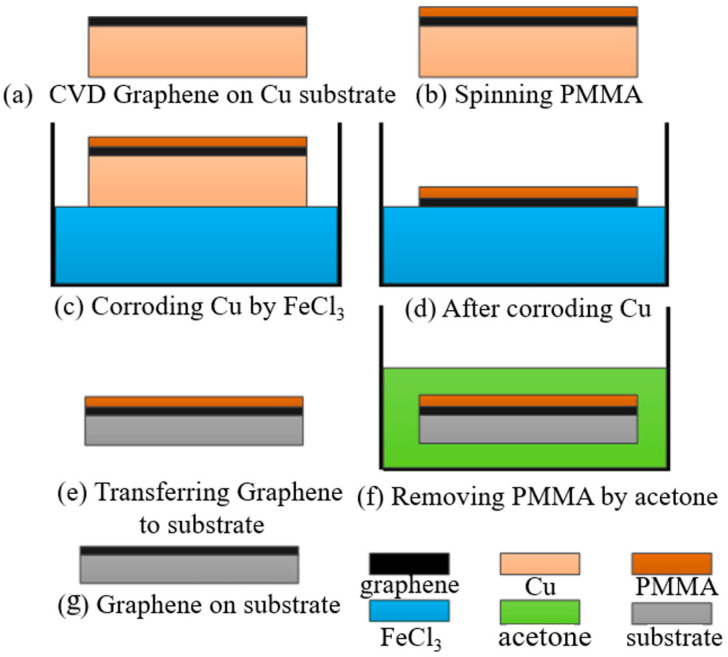
Wet transfer process of graphene deposition.

The graphene film used in the experiment was prepared by the CVD method and deposited on copper. The PMMA-assisted wet transfer method was adopted. Firstly, 4 g of PMMA powder was weighed and placed in a brown, wide-necked bottle. Then, 100 mL of benzaldehyde solution was poured into the bottle, and the bottle was heated in a water bath at 50 °C and magnetically stirred for 6 h. The PMMA was prepared by spin coating onto the graphene surface by a homogenizer and was heated and cured at 180 °C for 3 min. Next, 1 mol/L of FeCl_3_ solution was prepared. Due to its ultra-thin characteristic, graphene will quickly adsorb impurities, such as molecules and particles, in an aqueous solution. So, a dust-free cloth was used to filter the impurities on the solution surface. The residual FeCl_3_ contaminants in the graphene film were neutralized by 10–20%-diluted hydrochloric acid for 1 h and then further cleaned with deionized water. The substrate was washed with deionized water and acetone successively, and deionized water was finally used to reduce ion pollution. Then, the graphene/PMMA was transferred to the substrate and let stand to naturally dry. Finally, the PMMA was removed by acetone to complete the transfer process of graphene film. The specific steps of the operation are shown in Figure 1.

**Figure 2:**
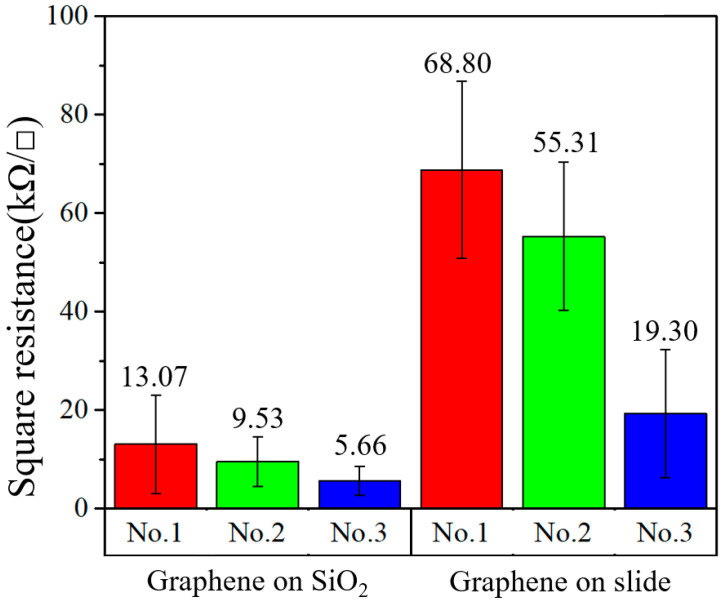
Square resistance of graphene on different substrates.

Graphene was transferred to glass slides and SiO_2_ substrates. On different substrates (SiO_2_ and glass), three groups of samples, named “No.1”, “No.2” and “No.3”, were prepared. Using an HPS2661 four-probe resistance measuring instrument (produced by Helpass Electronic Technologies), each sample was measured five times and its square resistance was indicated by an error bar. The test results of the different substrates are shown in Figure 2. It was found that the resistances between the same kind of substrate were significantly different, and the minimum resistance values of different substrates were several times different. As shown in Figure 1, on the slide, the minimum resistance of graphene was 19.30 kΩ/□ and the maximum resistance was 68.80 kΩ/□; on the SiO_2_ substrate, the minimum square resistance was 5.66 kΩ/□ and the maximum square resistance was 13.07 kΩ/□. It can be concluded that the square resistance of the graphene transferred to the SiO_2_ substrate was lower than that transferred to the glass substrate. In addition, the square resistances of various samples on like substrates were different.

The square resistance of graphene on a slide and on SiO_2_ is different because of the impurities and defects produced during the transfer process, though surface roughness may also be one of the influencing factors. In order to clarify the reasons for the differences in square resistance, surface roughness, number of layers and impurities were analyzed by AFM, a surface tension instrument, XPS and Raman spectrum analysis.

## 3. Results and Discussion

### 3.1. Influence of Surface Roughness on the Square Resistance of the Transferred Graphene

**Figure 3:**
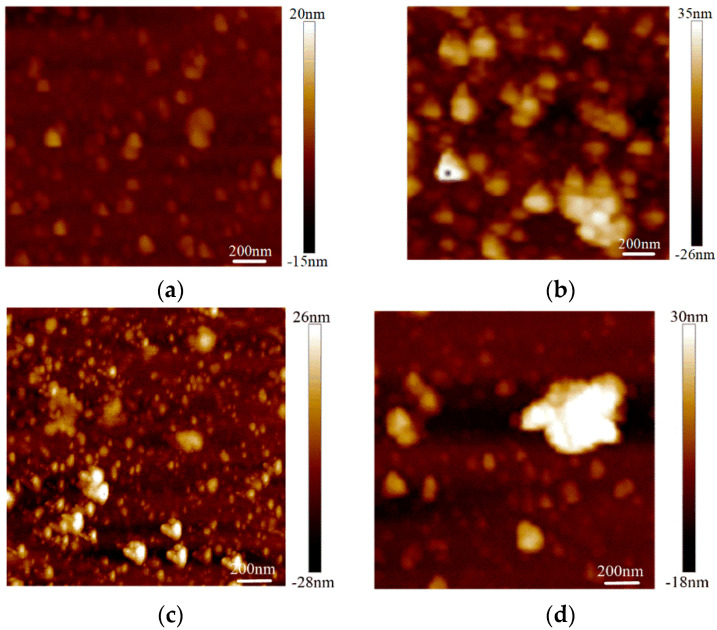
Surface roughness of graphene on a SiO_2_ substrate by AFM mapping. (**a**) Roughness of bare SiO_2_; (**b**) roughness of the No.1 sample on SiO_2_ (13.07 kΩ/□); (**c**) roughness of the No.2 sample on SiO_2_ (9.53 kΩ/□); (**d**) roughness of the No.3 sample on SiO_2_ (5.66 kΩ/□).

**Figure 4:**
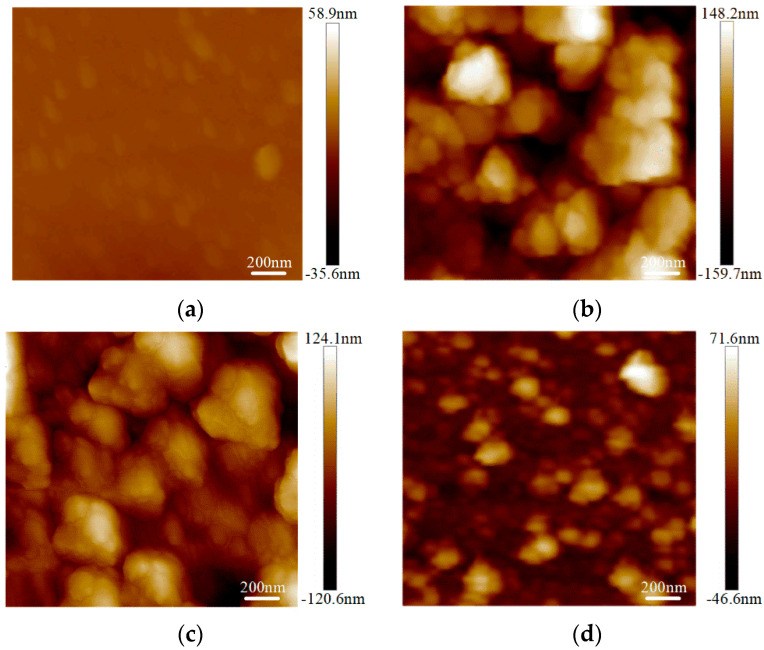
Surface roughness of graphene on a glass slide by AFM mapping. (**a**) Roughness of bare glass slide; (**b**) roughness of the No.1 sample on a glass slide (68.80 kΩ/□); (**c**) roughness of the No.2 sample on a glass slide (55.31 kΩ/□); (**d**) roughness of the No.3 sample on a glass slide (19.30 kΩ/□).

The surface roughness tests were conducted on the graphene on the SiO_2_ substrate by AFM with an Innova-IRIS atomic force microscope (Bruker Nano Surfaces co., Ltd., Billerica, MA, USA). Bare SiO_2_ was used as a blank control group in the experiment. As shown in Figure 3, there were almost no noticeable particles on the bare SiO_2_ substrate. In the No.3 sample on the SiO_2_ substrate particles were evenly distributed and its flatness was average. There were some large particles in the No.2 sample on the SiO_2_ substrate. For the No.1 sample, the surface was more uneven. The surface roughness was obtained with the NanoScope analysis software. The surface roughness, expressed as Ra),of the bare SiO_2_ substrate was 1.23 nm, the Ra of the No.3 sample on the SiO_2_ was 4.65 nm and the Ra of the No.2 and No.1 samples on SiO_2_ were 6.71 nm and 7.86 nm, respectively. From Figure 4, we can see that the surface roughness of the glass substrate was similar to that of the SiO_2_ substrate. The surface roughness of the sample with the lowest square resistance (Figure 4d) was less than the other two experimental samples. The Ra of the bare glass slide was 25.68 nm. The Ra of the No.1, No.2 and No.3 samples were 70.14 nm, 58.23 nm and 30.26 nm, respectively.

It can be concluded, first, that the more severe the surface roughness, the greater the square resistance of the transferred graphene, and, second, that surface roughness was related to surface pollution.

### 3.2. Influence of Surface Impurity on the Square Resistance of the Transferred Graphene

#### 3.2.1. Indirect Measurement by Surface Contact Angle

Graphene has an enormous specific surface area because of its atomic layer structure. Therefore, it is easy to adsorb various impurities in the experimental process, which will severely impact the electrical properties of graphene. It usually adsorbs H_2_O, O_2_, NO_2_ or other molecules when exposed to air and can form P-type doping with H_2_O and NO_2_, opening the bandgap of graphene [13]. When graphene absorbs H_2_O, the particular charge of the latter is transferred, and a reverse bias voltage is formed, like a diode between the graphene and water molecules. Therefore, the binding energy with water increases, showing a smaller contact angle. In addition, a small amount of PMMA causes p-type doping with graphene [20,21], which forms a competitive relationship with H_2_O and doped graphene, resulting in a smaller binding force and larger contact angle.

**Figure 5:**
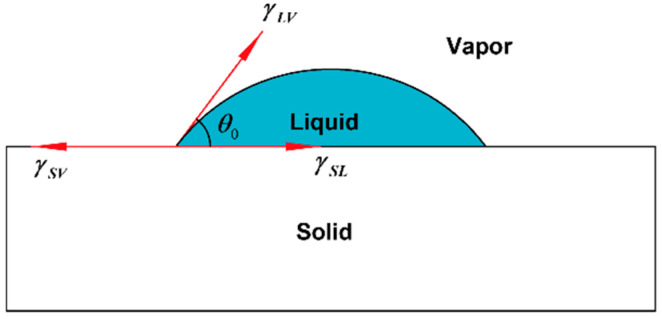
Schematic diagram of liquid contact angle.

**Figure 6:**
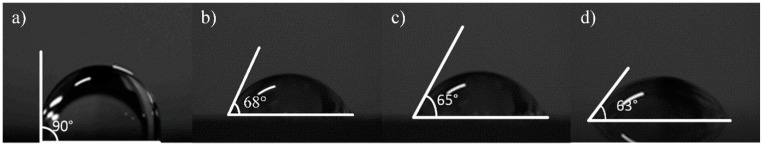
The inclination angle of deionized water from different graphene samples on SiO_2_, measured by a surface tension instrument; (**a**) on SiO_2_ without graphene; (**b**) the No.1 sample on the SiO_2_ substrate (13.07 kΩ/□); (**c**) the No.2 sample on the SiO_2_ substrate (9.53 kΩ/□); (**d**) the No.3 sample on the SiO_2_ substrate (5.66 kΩ/□).

**Figure 7:**
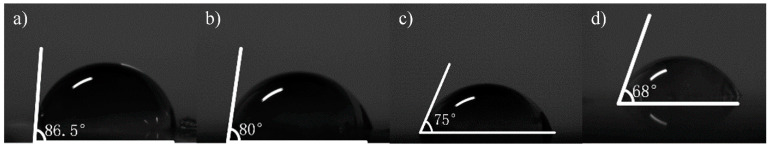
The inclination angle of deionized water from different graphene samples on slides, measured by a surface tension instrument; (**a**) on a slide without graphene; (**b**) the No.1 sample on the slide substrate (68.8 kΩ/□); (**c**) the No.2 sample on the slide substrate (55.31 kΩ/□); (**d**) the No.3 sample on the slide substrate (19.3 kΩ/□).

Contact angle measurement can characterize the quality of graphene transferred to a surface. In this work, a pipette with a specification of 0.1–2.5 µL was used to collect deionized water, and the volume of water drops therefrom was 0.1 µL. Then, the deionized water dip angles were measured for the graphene of the No.1 (13.07 kΩ/□), No.2 (9.53 kΩ/□) and No.3 (5.66 kΩ/□) samples transferred to the SiO_2_ substrate, which were measured qualitatively through a JC2000 contact Angle analyzer (Shanghai Zhongchen Digital Technic Apparatus Co., Ltd., Shanghai, China). As shown in Figure 5, the dip angles of the left and right sides of the droplets were measured, and the formula for calculating contact angle is shown in Formula 1 [33]. Then the average value was calculated to eliminate error caused by the non-horizontality of the table. The dip angle of the droplets on the SiO_2_ substrate was 90°, the dip angle of the No.1, No.2 and No.3 samples on the SiO_2_ substrate were 68°, 65° and 63°, respectively (Figure 6). While the dip angle of the droplet on the slide substrate was 86.5°, the dip angle of the No.1, No.2 and No.3 samples on the slide substrate were 80°, 75° and 68°, respectively (Figure 7).

(1) $$\gamma_{SV}=\gamma_{SL}+\gamma_{LV}\times \cos\theta_{e}$$

The contact angles of the samples on the SiO_2_ and slide substrates were different because the surface defects reduced the binding forces. The defect content of each sample was also different, as were the contact angles. Next, XPS was used to determine the kinds of residues or impurities on the graphene surfaces.

#### 3.2.2. Element and Composition Measurement by XPS

**Figure 8:**
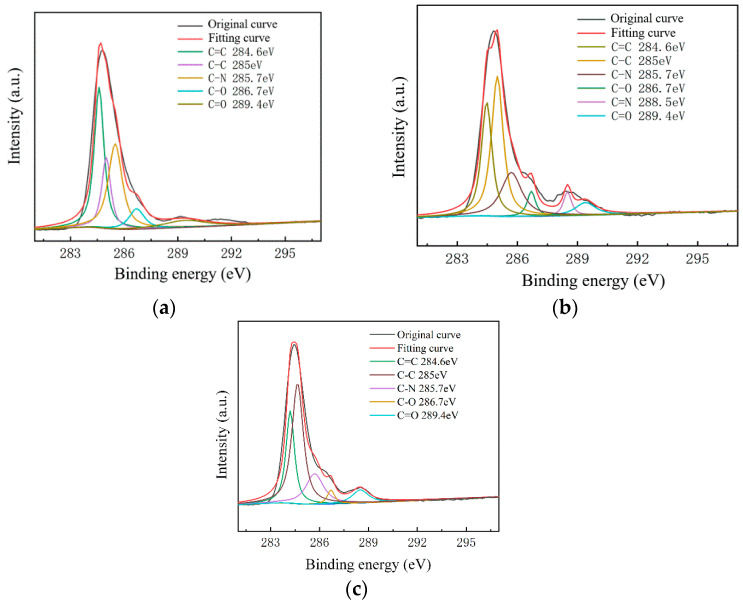
C1s peak separation results of samples on the slide substrate; (**a**) the No.1 sample 68.80 kΩ/□); (**b**) the No.2 sample (53.31 kΩ/□); (**c**) the No.3 sample (19.30 kΩ/□).

**Figure 9:**
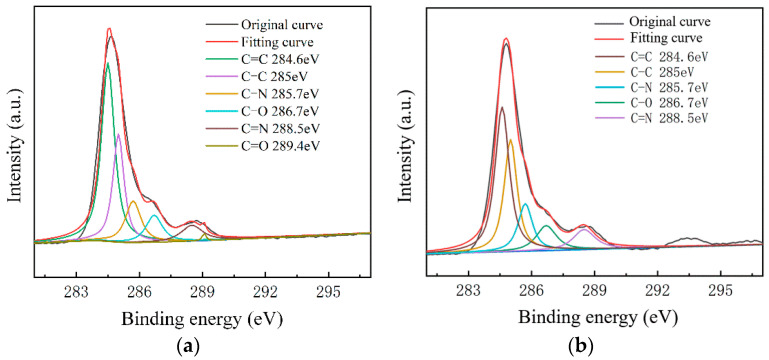

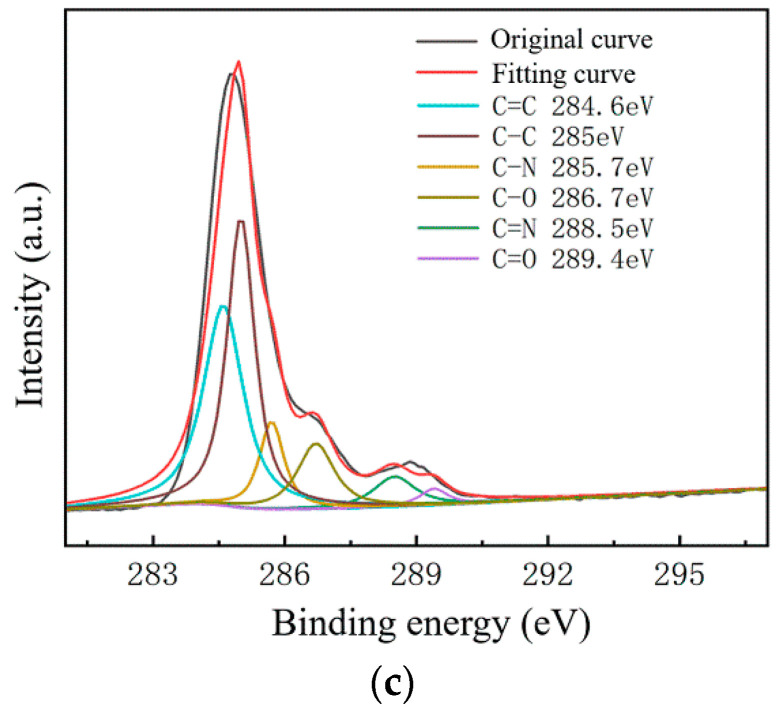
C1s peak separation results of samples on the SiO_2_ substrate; (**a**) the No.1 sample (13.07 kΩ/□); (**b**) the No.2 sample (9.53 kΩ/□); (**c**) the No.3 sample (5.66 kΩ/□).

To further study the influence of graphene surface impurities on the electrical properties of the obtained graphene, it was necessary to analyze the graphene surface in the X-ray energy spectrum, for which we used an ESCALAB Xi^+^ X-ray photoelectron spectroscopy (Thermo Fisher). The contents of the elements C, N and O on the surfaces of the experimental samples after transfer were measured by XPS analysis, and information of the various elements’ peaks was obtained. The C1s peak is the most significant peak for the analysis of doping substances. Therefore, the C1s peak was processed by XPS peak separation. The chemical bond composition of C in the C1s peak was analyzed as the baseline for introducing impurity species during the graphene transfer process. Different substrate samples are selected for analysis, as shown in Figure 8 and Figure 9.

As shown in Figure 7, The C–N content and C–O content were almost unchanged. For the samples on slide substrates, as the content of C–C increased, the content of C=C decreased. The C=C content of the No.1, No.2 and No.3 samples was 26%, 16% and 13%, respectively. As shown in Figure 8, the transferred graphene on the SiO_2_ substrate had a similar trend. On the same substrate, samples of greater square resistance had more C=C content. No matter the substrate, only PMMA molecules contained a large amount of C=C. PMMA, as an impurity, when doped with graphene, opens the graphene’s bandgap [13,27,31,34].

Combined with the contact angle test results, it was found that the greater the square resistance of the same group of samples, the greater the contact angle. It was speculated that the substance on the surface of the sample affects the surface binding energy of deionized water. XPS analysis results showed that a large amount of C=C remained on the substrate surfaces, and only PMMA contained C=C during the experiment. Compared with the water contact angle test and XPS results (Figure 6, Figure 7, Figure 8 and Figure 9), it was found that the less the PMMA contamination, the smaller the water contact angle, consistent with the reported work [35,36]. Additionally, PMMA can cause p-type doping with graphene [20,21], leading to a competitive relationship with H_2_O and doped graphene, in turn resulting in a smaller binding force and larger contact angle. Therefore, PMMA is the substance with the greatest influence on the square resistance because it can be doped with graphene and thereby open the graphene bandgap.

### 3.3. Influence of Layer Lamination and Cracking on the Square Resistance of the Transferred Graphene

**Figure 10:**
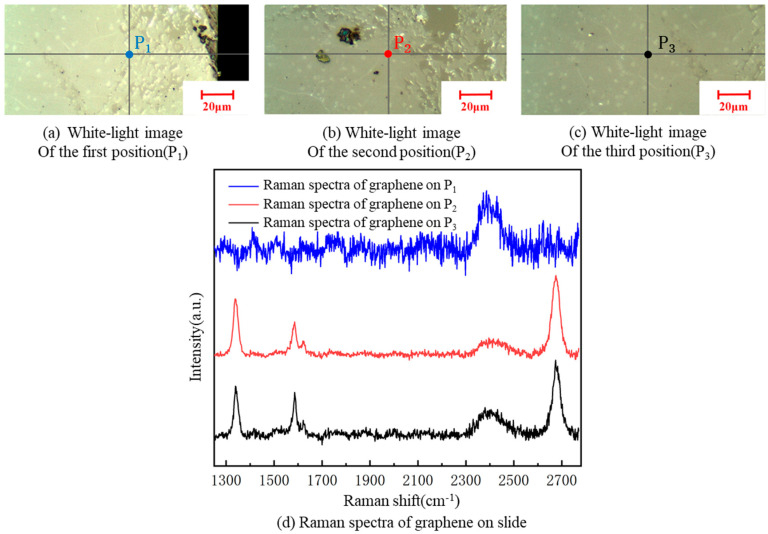
A white-light image of three different positions on the slide substrate (**a**–**c**). Raman spectra of graphene at the three positions on the slide (**d**).

A Renishaw inVia Qontor laser confocal Raman spectrum system with a 532-nm wavelength was used in the experiment. The Raman spectrum of graphene has two characteristic peaks: G and 2D peaks. The G peak (usually around 1580 cm^−1^) is the characteristic peak of the sp^2^ structure, reflecting the symmetry and crystallinity of graphene, which is generated by the double-degenerate iTO and iLO optical phonon interactions in the center of the Brillouin zone, with E2g symmetry. The 2D peak (usually around 2700 cm^−1^) is generated by two inter-valley inelastic scatterings of iTO optical phonons near point K, which is used to characterize the stacking mode of the carbon atoms in graphene. In addition, the D peak and D’ peak reflect defects. If the laser is focused on a graphene samples containing defects or its grain boundary, a defect peak D, at about 1350 cm^−1^, and a peak D’, at about 1620 cm^−1^, appear. The number of graphene layers can be determined by the ratio of I_2D_/I_G_. If I_2D_/I_G_ is two, graphene is of a single layer.; if I_2D_/I_G_ is one, it is bilayered. In practice, the ratio of I_2D_/I_G_ is generally not exactly equal to two. In such case, we can judge the number of graphene layers by its Raman feature. As there is often a multi-layer core at the center of each flake, when it is hard to judge graphene as single- or double- layered, a blue shift in the 2D peak of graphene can be used as the basis for evaluating double layers [37]. A sample, with a resistance 68.80 kΩ/□, of graphene was taken. Raman spectroscopy detection of graphene films at three different positions on each kind of substrate was carried out. The Raman spectra of the three measuring points of graphene on the glass slide showed defect peaks D and G‘, indicating some defects in the transferred graphene to the glass slide. It can be clearly seen from Figure 10 that the I_2D_/I_G_ was less than one, indicating that the graphene stacking phenomenon was serious, and the number of graphene layers increased to various degrees after the transfer; thus, the square resistance significantly improved [24].

**Figure 11:**
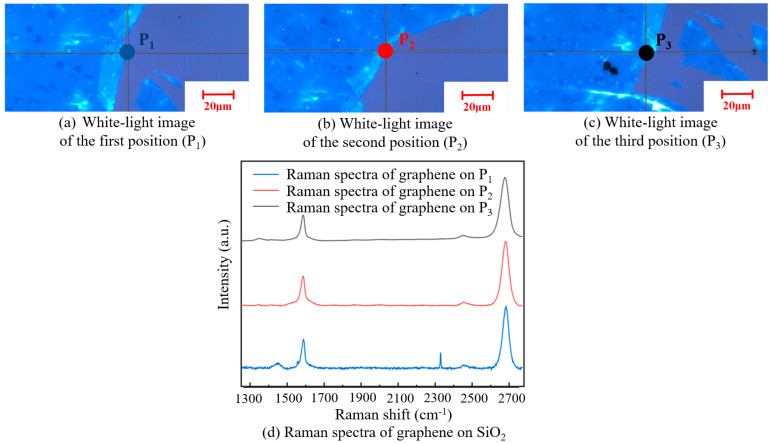
A white-light image of three different positions on the SiO_2_ substrate (**a**–**c**). Raman spectra of graphene at the three positions on the SiO_2_ substrate (**d**).

Figure 11 shows a white-light image of graphene with a square resistance of 5.66 kΩ/□) on the SiO_2_ substrate and its Raman spectra. According to the Raman spectra of the three tested positions, I_2D_/I_G_ was about two, indicating that the graphene was monolayered and not destroyed after transfer. The number of graphene layers was maintained with good uniformity. However, it can be seen from the Raman spectrum of the first position that there was a defect peak D, indicating that a small number of defects appeared on the surface.

According to Figure 10, the graphene was cracked, single-layered and double-layered, with poor uniformity at the three graphene positions, P_1_, P_2_ and P_3_, on the slide substrate, respectively. There were defect D peaks in all of them. The square resistance of graphene on the slide was more significant, overall, than on the SiO_2_ substrate, because the square resistance of graphene is proportional to the number of layers [24].

## 4. Conclusions

In conclusion, the defects that occurred in the wet transfer process greatly influenced the electrical properties of the produced graphene. Defects, such as PMMA residue, delamination and cracking during the wet transfer process, were discovered with various instruments. AFM imaging found that, on the same substrate, samples’ roughness and square resistance increased. The samples with higher square resistance had larger water contact angles and more PMMA content. As PMMA residue can cause a smaller contact angle due to a competitive relationship with H_2_O and graphene; it can nonetheless widen graphene’s bandgap, leading to more significant square resistance. Therefore, PMMA affected the square resistance of the samples seriously. Additionally, there were stacks in the samples, observed by their Raman spectra, but they were not the main factor in the square resistances of the transferred graphene samples. This study provided a theoretical and experimental basis for graphene as a better electrical medium.
